# Screening for inhibitors of mutacin synthesis in *Streptococcus mutans* using fluorescent reporter strains

**DOI:** 10.1186/s12866-018-1170-3

**Published:** 2018-03-27

**Authors:** Priyanka Premnath, Michael Reck, Kathrin Wittstein, Marc Stadler, Irene Wagner-Döbler

**Affiliations:** 1 Helmholtz-Center for Infection Research, Group Microbial Communication, Inhoffenstr. 7, 38124 Braunschweig, Germany; 2 Helmholtz-Center for Infection Research, Department of Microbial Drugs, Inhoffenstr. 7, 38124 Braunschweig, Germany

**Keywords:** *Streptococcus*, Virulence, Mutacin, Whole-cell screening, Fluorescent reporter, Myxobacteria, Fungi, Secondary metabolites

## Abstract

**Background:**

Within the polymicrobial dental plaque biofilm, bacteria kill competitors by excreting mixtures of bacteriocins, resulting in improved fitness and survival. Inhibiting their bacteriocin synthesis might therefore be a useful strategy to eliminate specific pathogens. We used *Streptococcus mutans*, a highly acidogenic inhabitant of dental plaque, as a model and searched for natural products that reduced mutacin synthesis. To this end we fused the promoter of mutacin VI to the GFP+ gene and integrated the construct into the genome of *S. mutans* UA159 by single homologous recombination.

**Results:**

The resulting reporter strain 423p - *gfp +* was used to screen 297 secondary metabolites from different sources, mainly myxobacteria and fungi, for their ability to reduce the fluorescence of the fully induced reporter strain by > 50% while growth was almost unaffected (> 90% of control). Seven compounds with different chemical structures and different modes of action were identified. Erinacine C was subsequently validated and shown to inhibit transcription of all three mutacins of *S. mutans.* The areas of the inhibition zones of the sensor strains *S. sanguinis* and *Lactococcus lactis* were reduced by 35% to 61% in comparison to controls in the presence of erinacine C, demonstrating that the amount of active mutacins in the culture supernatants of *S. mutans* was reduced. Erinacines are cyathane diterpenes that were extracted from cultures of the edible mushroom *Hericium erinaceus.* They have anti-inflammatory, antimicrobial and neuroprotective effects. For erinacine C, a new biological activity was found here.

**Conclusions:**

We demonstrate the successful development of a whole-cell fluorescent reporter for the screening of natural compounds and report that erinacine C suppresses mutacin synthesis in *S. mutans* without affecting cell viability.

**Electronic supplementary material:**

The online version of this article (10.1186/s12866-018-1170-3) contains supplementary material, which is available to authorized users.

## Background

The lack of novel antibiotics in the pipeline of pharmaceutical companies and the simultaneous worldwide increase of antibiotic resistance represent enormous challenges in the re-emerging fight against infectious diseases [1]. In addition, the discovery of the complex functions of the human microbiome in maintaining health and its crucial contribution to the development of the immune system in infants call for completely new strategies [2]. As an alternative to killing the pathogens, quenching their quorum sensing mechanisms has been explored because resistance is expected to be very slow or unlikely. This is because the strategy does not interfere with growth and only changes its phenotype with minimal or no selective pressure [3–7]. For example, in *P. aeruginosa*, the cell-cell communication required for establishing virulence was targeted. Mimics of its quorum sensing signal N-(3-oxododecanoyl)-L-homoserine lactone which were structurally similar to the natural AHL but unable to induce quorum sensing were synthesized and competed for the natural AHL resulting in reduced production of the virulence factor pyocyanin [8].

In this context, interest in compounds synthesized by bacteria, fungi, plants and animals has increased [9]. Myxobacteria are a particularly rich source of secondary metabolites [10] and have recently been found to yield various novel chemical entities that appear promising as starting points for development of novel antibiotics [11–13]. Natural compounds have unique, highly complex chemical structures and dedicated biosynthetic pathways and have been optimized in evolution for improving the survival of the producing organism; their biological function is therefore context dependent and often not known [14]. In particular from fungi, numerous novel unique metabolites are continuously being discovered, which may serve as basis for novel lead structures to combat bacterial pathogens [15]. For example, caspofungin, a semi-synthetic derivative of pneumocadin from *Glarea lozoyensis*, was active against *Candida albicans* biofilms and was the first fungal inhibitor that has been approved for treatment against invasive candidiasis [16]. Aspergillomarasmine A (AMA), from *Aspergillus versicolor* was recently identified to inhibit the function of metallo-β- lactamase NDM-1 of Enterobacteriaceae. It sequestered the Zn2+ required activity of NDM-1 metalloproteinase, which opens the β-lactam ring of the antibiotic thereby causing resistance. However, AMA with its sequestration ability re-sensitizes bacteria to meropenem [17].

Here we focused on *Streptococcus mutans*, an important component of dental plaque. *S. mutans* was considered the etiological agent of caries based on cultivation-dependent analyses [18]. Current next generation sequencing approaches found *S. mutans* to be a minor component of caries lesions and revealed the contribution of many more oral species to caries development [19–22]. In fact *Veillonella* sp. was identified to be predictive of early childhood caries, but not *S. mutans* [23]. Here we use *S. mutans* as a model and test a general strategy that might be transferable to other streptococci. For this aim, *S. mutans* is ideally suited because it is very well studied and many genetic tools are available.

Here we now targeted the ability to produce mutacins, the peptide antibiotics synthesized by *S. mutans* [24]. According to their structure, they are divided into non-lantibiotics and lantibiotics, of which the lantibiotic mutacin 1140 has attracted large interest as anti-bacterial drug [25, 26]. Previously a strain of *S. mutans* was developed that produced elevated levels of mutacin 1140 while being impaired in acid production [27, 28]. It was planned to be applied as a probiotic to outcompete wild-type *S. mutans* for the so-called replacement therapy of dental caries and underwent phase 1 clinical testing [29].

Here we pursued an alternative approach. Since mutacins kill competitors [30, 31], suppression of their synthesis should impair the fitness of *S. mutans* in the polymicrobial dental plaque biofilm. Natural compounds that suppress mutacin production might be more easily approved by the authorities than a genetically engineered probiotic. Therefore we developed whole-cell reporters of *S. mutans* UA159 where we fused the promoters of mutacin synthesis genes to the enhanced GFP+ and monitored fluorescence as a proxy of gene expression. Mutacin synthesis in *S. mutans* is under the control of the autoinducer MIP (mutacin inducing peptide) [32]. Thus we induced mutacin synthesis by MIP and monitored reduction of fluorescence in the presence of test compounds. In such a way we screened 297 natural compounds from myxobacteria and fungi, of which the most interesting compound was subsequently validated.

## Methods

### Bacterial strains and cultivation conditions

*Escherichia coli* DH5α was grown aerobically in Luriani Bertani (LB) broth (Carl Roth, X969.2) at 37 °C. Selection of positive clones in *E. coli* was done on LB plates with 1.5% agar and 300 μg/ml erythromycin (Sigma Aldrich, E5389). Overnight cultures of *Streptococcus mutans* UA159 (ATCC 700610) were routinely grown in Todd Hewitt broth supplemented with 0.5% (*w*/*v*) yeast extract (THBY; Becton Dickinson, 249,240, 212,750) at 37 °C and 5% CO_2_ without shaking. 10 μg/ml of erythromycin was used for selection of positive clones on THBY agar. The indicator strains *L. lactis* for SMU_1914 and *S. sanguinis* for SMU_150 were routinely grown in THBY broth at 37 °C and were overlaid with 0.7% THBY soft agar in the mutacin overlay assay. For screening, the mutacin expression reporters were grown on a semi-defined BM medium [33] where the concentration of glucose was increased to 0.5%. BM medium was chosen because it had minimal background fluorescence.

### Construction of fluorescent reporter strains

Plasmid pAE03 [34] was used for the construction of the mutacin expression reporters. The plasmid encodes an erythromycin cassette for selection and a promotorless-GFP+ fluorophore to which the respective mutacin promotors were fused. The promotors of the mutacin genes SMU.423 (mutacin VI), SMU.150 (mutacin IV) and SMU.1914 (mutacin V) were amplified using fusion polymerase (New England Biolabs, M0530S) and the primers listed (see Additional file 1). PCR products were cloned into the plasmid pAE03 using the Clone EZ Genscript kit (L00339). The recombination reaction was carried out in a thermocycler at 22 °C for 30 min. The mixture was transformed into *E.coli* DH5α competent cells and clones were selected on THBY agar with 300 μg/ml erythromycin (Sigma Aldrich, E5389). From the positive clones, plasmids were purified, transformed into *S. mutans* UA159 and selected on THBY agar with 10 μg/ml of erythromycin. Positive clones where promoter and GFP+ were fused by single homologous recombination were confirmed by PCR and sequencing.

### Construction of deletion mutants in *S. mutans*

The mutacin genes were amplified from genomic DNA of *S. mutans* with specific primers (see Additional file 1). 5′ end of the forward and reverse primer for erythromycin, and P2/P3 sequences of the gene amplification primers were incorporated with restriction sites (*Asc*I and *Fse*I) to create overhangs for ligation. The amplified sequences were digested with the restriction enzymes *Ase*I and *Fse*I (New England biolabs, R0558S, R0588S). The digested fragments were purified using QIAquick PCR purification kit (28106). The concentration of the fragments was determined using nanodrop and confirmed by agarose gel electrophoresis. The digested erythromycin cassette and the flanking regions were ligated with T4 DNA ligase (New England biolabs, M0202). The ligation reaction was carried out at RT for 10 min followed by inactivation at 65 °C for 10 min in a thermocycler. The ligation product was transformed into *S. mutans* UA159 and positive clones were selected by 10 μg/ml of erythromycin. Deletion constructs were verified by PCR and sequencing.

### Transformation in *E. coli* and *S. mutans*

Transformation in *E. coli*: SOC medium was thawed at 37 °C and TSS solution was placed on ice. An overnight culture of *E. coli* DH5α was diluted 1:100 in 5 ml sterile LB broth and incubated at 37 °C at 200 rpm until an OD 0.3–0.4. 1 ml of the culture was centrifuged (13,000 rpm, 1 min), the supernatant was discarded and the pellet re-suspended in 100 μl ice-cold TSS solution. The plasmid construct (5–10 μl) was added to the cells and incubated on ice for 30 min followed by a heat shock at 42 °C for 45 min with subsequent transfer to ice for 2 min. The transformation mixture was transferred to 600 μl SOC medium and incubated for 1 h in the 37 °C shaker. The cells were centrifuged (10,000 rpm, 2 min) and 550 μl of the SOC medium was removed. The pellet was re-suspended in the residual medium and plated on LB agar with antibiotics when needed. The plates were incubated at 37 °C overnight. Positive colonies were picked, cultivated in LB, DNA was isolated and the construct was verified by sequencing.

Transformation in *S. mutans*: An overnight culture of *S. mutans* was diluted 1:10 in 5 ml THBY medium. The cells were incubated at 37 °C and 5% CO_2_ for 2 h. 5 μl of 1 mM MIP was added and further incubated at 37 °C for 30 min. 200 μl of the culture was placed in an Eppendorf tube, DNA was added and the cells were incubated for 3 h. Selection of constructs was performed on THBY agar with the respective antibiotics. Positive clones were picked, cultivated in THBY, DNA was isolated and the constructs were verified by sequencing.

### Screen for suppression of mutacin expression

The mutacin reporter strains (150p, 1914p and 423p with *gfp+*) were cultivated in THBY broth with 10 μg/ml erythromycin overnight. 15 ml of the cultures were harvested by centrifugation (Thermo scientific X1R, 6000 rpm, 15 min, 4 °C). The supernatant was decanted and pellets were gently re-suspended in 7 ml of freshly prepared BM-glucose medium using a pipette. The OD_600_ of the cultures was adjusted to 0.2 using the Ultrospec 3100 pro U*V*/VIS spectrophotometer.

The assay was performed with a final culture volume of 200 μl per well on a 96 well optical bottom polystyrene black microtitre plate (Thermo Scientific, 165305). 0.5 μl of 1 mM mutacin inducing peptide (MIP) synthesized in-house and 10 μl of the test compounds solved in methanol or DMSO were added to each well. Wells with MIP but without test compound served as controls. Plates were incubated at 37 °C, 5% CO_2_, without shaking, for 4 to 24 h. Absorbance (P620/8 nm filter) and fluorescence (485_nm_excitation/535 _nm_ emission) were determined with a plate reader (Perkin Elmer, Victor^3^1420 Multilabel Counter). For the KOM and MWIS libraries, the assay was performed in triplicate, the SAAR plates contained each compound once. Data are mean and standard deviation from two independent experiments for the KOM and MWIS libraries. Validation experiments were performed in triplicate assays and two biological replicates.

### Natural product libraries

A total of 297 compounds were screened and were derived from three different natural product libraries which are named after the groups that provided them KOM (Microbial Communication, HZI, Germany), MWIS (Microbial Drugs, HZI, Germany) and SAAR (Helmholtz-Institute of Pharmaceutical Research, Saarbrücken, Germany).

In case of KOM and MWIS, the stocks of the compounds were provided in methanol. These libraries were screened at a final concentration of 5 μg and 0.5 μg per ml in triplicates. Compounds from SAAR were provided on a polypropylene micro-titre plate with 5 μl of the compound at 1 mM concentration in DMSO. Plates were stored at − 80 °C. Shortly before screening, the plate was retrieved from − 80 °C and thawed at room temperature. To pool the compounds to the bottom, the plate was centrifuged in a microplate centrifuge (Thermo scientific Heraeus labofuge 400 R) at 1500 rpm for 5 min and the compounds were transferred to an optical bottom polystyrene black microtitre plate for testing. The final concentration of the compounds from the SAAR library was 25 μM.

### Mutacin overlay assay

Overnight cultures in THBY of *S. mutans* UA159 and the deletion mutants Δ150, Δ1914 and Δ423 were adjusted to OD_600_ of 0.2. To each culture erinacine C (5 μg/ml) was added, incubated (45 min) and then centrifuged (7500 rpm, 5 min). The supernatant was discarded and the pellet was re-suspended in 50 μl fresh THBY. 2.5 μl of each sample was spotted on THBY agar. The plates were incubated for 4–6 h. Afterwards, 2.5 μl of 1 mM MIP and 2.5 μl of 0.2 mg/ml erinacine C was added to the spot and incubated overnight. On the following day, overnight cultures of *L. lactis* and *S. sanguinis* were adjusted to an OD_600_ of 0.1 and cultivated for 2.5 h. After 2.5 h, 100 μl of the mid-log phase cultures was diluted in 6 ml of 0.7% THBY top agar and overlaid. After 20 h incubation, the area of inhibition around each spot was measured.

## Results

### The mutacin VI reporter shows strong population wide expression upon induction by MIP

**Fig. 1:**
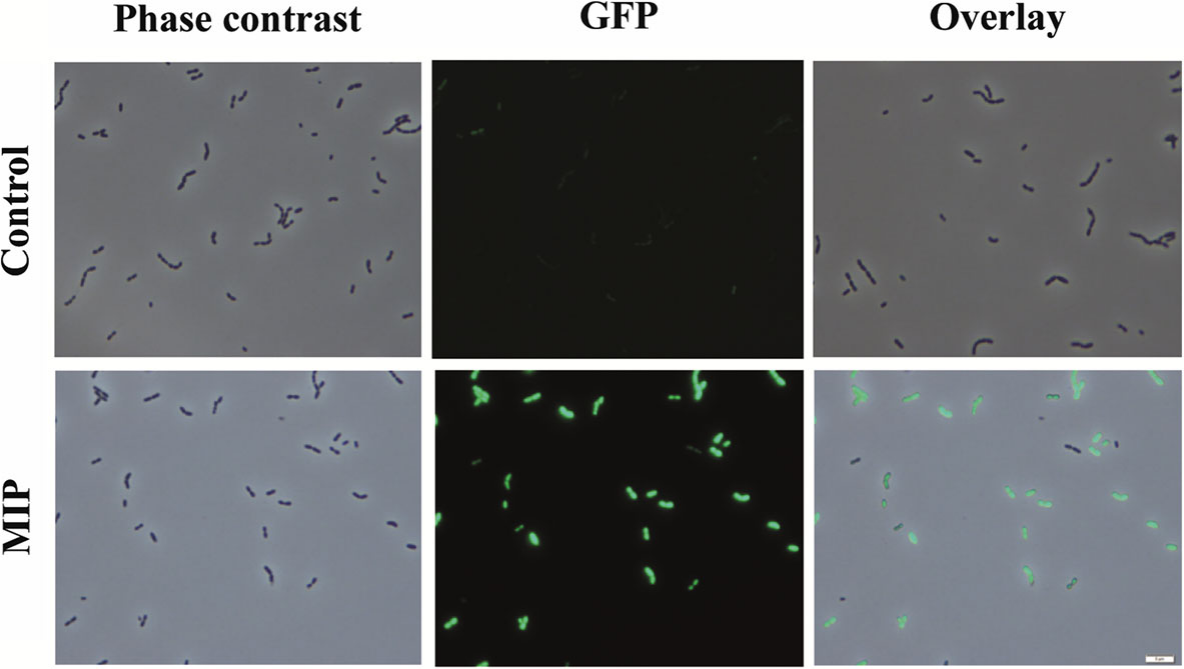
Population wide activation of the mutacin VI promoter of *S. mutans* UA159 by MIP. Reporter strain *S. mutans* 423p – *gfp +* was cultivated in BM medium supplemented with 0.5% glucose and 2.5 μM MIP (for induction of mutacin transcription). After 8 h of growth (37 °C, 5% CO_2_) cells were observed under the fluorescence microscope. See Methods for further details

*S. mutans* produces the three non-lantibiotic mutacins IV, V and VI. Mutacin IV, which is also termed NlmAB, acts against many streptococcal strains, while mutacin V (also termed NlmC or CipB) is active against non-streptococcal species [35]. The activity of mutacin VI was only detected in a strain overexpressing the novel regulatory system BrsRM [36, 37] and thus no natural indicator strain is known. In spite of this lack of knowledge, we initially used the mutacin VI (SMU.423) reporter for screening because natural compounds might have elicited strong responses and this would have been an important novel finding. The fully induced reporter construct showed the same growth as the wild-type (see Additional file 2) indicating that the expression of GFP+ did not represent a metabolic burden and should not affect screening results. As expected, transcription of mutacin VI occurred in a population wide manner in THBY upon induction by MIP (Fig. 1). Transcription of all mutacin genes is controlled by the response regulator ComE which is activated directly through the ComDE two-component system [32]. Deletion of the mutacin VI open reading frame had no influence on growth (see Additional file 3).

### Prescreening for inhibitors of mutacin VI transcription

Altogether 297 natural compounds were used for pre-screening the mutacin VI reporter strain. They contained fungal and myxobacterial secondary metabolites and were available as DMSO or methanol stocks, large fraction of which were provided in microtiter format at 25 μM [38]. Compounds were scored positive if they reduced the transcription of the maximally induced mutacin VI gene by more than 50% at three time-points without a strong effect on growth (OD_600_ > 90% of control induced by MIP but not treated by natural compound). Three time points were measured (t1, t2 and t3 in Table 1), namely at 4 or 6 h, 8 h and 24 h. The time points were chosen because mutacin production remains high during the onset of stationary phase or when the cell density reaches maximum. They represented exponential growth (between 4 and 6 h), onset of stationary phase (8 h) and stationary growth phase (> 8 h up to 24 h). Of the 297 compounds, 49 inhibited growth, and 18 showed strong background fluorescence. From the remaining 230 compounds, 7 were scored positive (Table 1). The strongest inhibitor was myxovirescin D which reduced reporter fluorescence to 13% of the control. Interestingly, variants of ambruticin, an anti-fungal compound [39] were able to increase the expression of the fully induced mutacin VI promoter even further (> 25%).

**Table 1:** Prescreening results for suppressors of mutacin VI transcription

Compound	% fluorescence			Structure	References
	t1	t2	t3		
Erinacine C	43	38	40		[47]
Noricumazol A	33	29	24		[42, 54, 55]
Myxovirescin D	26	18	13		[43]
Chondrochloren B	47	38	25		[56, 57]
Aurachin A	40	26	16		[44, 58, 59]
Myxopyronin A	23	20	27		[45, 60–62]
Jerangolid E	42	42	35		[46, 63]

Compounds that reduced fluorescence of the mutacin VI reporter at three time points (t1, t2, t3) by more than 50% without affecting growth (less than 10% reduction in OD_600_) are shown here. The total number of tested compounds was 297

### Erinacine C inhibits transcription of all three mutacins of *S. mutans* UA159

**Fig. 2:**
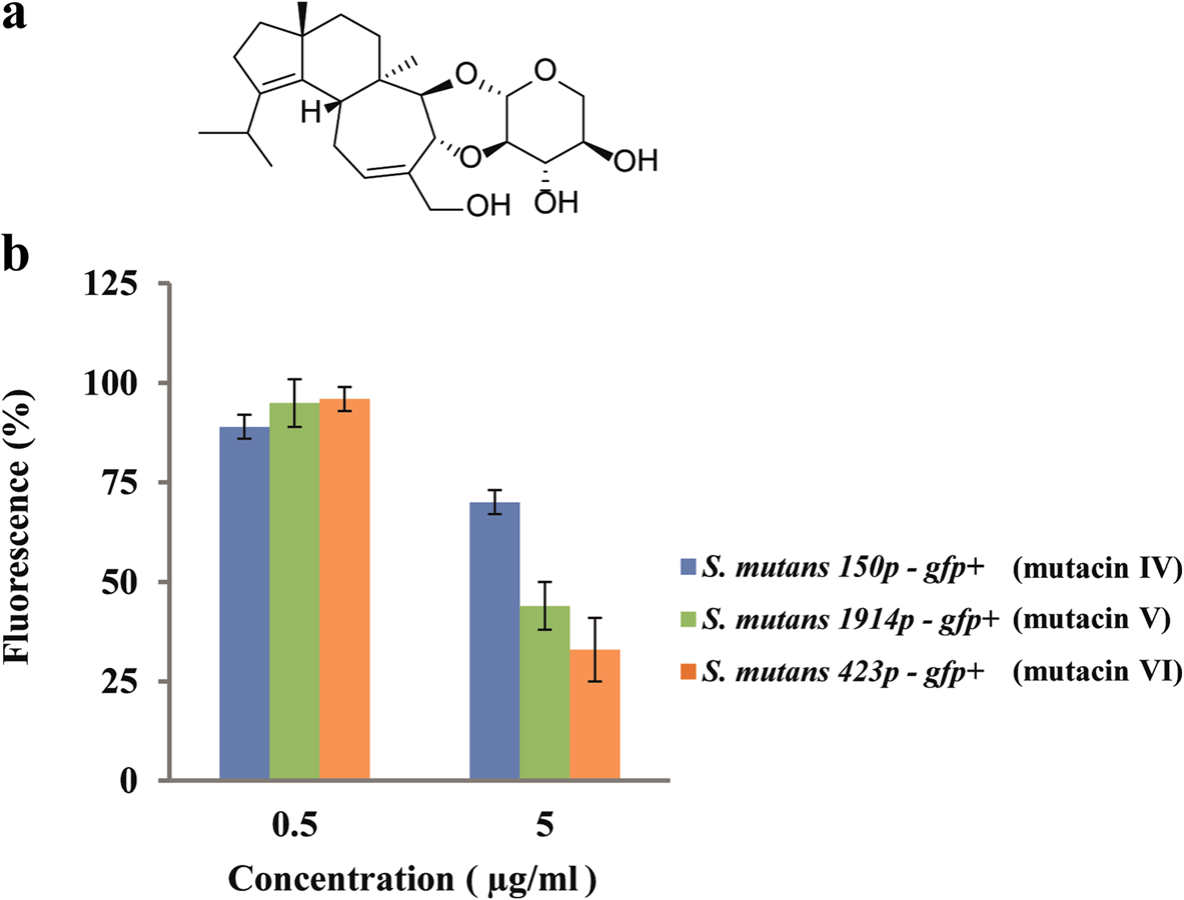
Reduction of mutacin IV, V and VI expression by erinacine C. Upper panel **a**: Chemical structure of erinacine C [47]. Lower panel **b**: Reporter strains 150p, 1914p and 423p (tagged with *gfp+*) were cultivated as in Fig. 1, except that erinacine C was added at the same time as MIP at 0.5 μg/ml or 5 μg/ml (final concentration). Data show mean and standard deviation of two biological replicates which were conducted with triplicate subsamples

From the seven compounds shown in Table 1 we chose erinacine C for further investigations. First, we tested if the results of the prescreening could be confirmed in laboratory culture. Then, we tested if this compound also suppressed the transcription of the two other mutacins of *S. mutans*. Mutacin IV is transcribed weakly and kills closely related Streptococci [35], while mutacin V is transcribed at much higher rates [32] and kills additionally *Lactococcus* species. To this end we used reporter strains 1914p – *gfp +* (for the promoter of SMU.1914, mutacin V) and reporter strain 150p – *gfp +* (for the promoter of SMU.150, mutacin IV). Figure 2 shows that at 5 μg/ml final concentration, erinacine C also reduced expression of mutacin IV and V, although the strength of inhibition varied. There was no growth inhibition at this concentration. Lower concentrations of erinacine C were inactive (Fig. 2, Additional file 4). At higher concentrations of erinacine C, growth was increasingly impaired (Additional file 4). At 20 μg/ml erinacine C, growth of the *S. mutans* reporter strains was reduced to approximately 50% of the controls.

### Erinacine C protects indicator strains from killing by *S. mutans*

Post-transcriptional regulatory mechanisms have a large influence on the expression of proteins and peptides, and play a particularly important role for the synthesis and maturation especially of bacteriocins. For example, in *S. mutans* transcription of mutacin V is induced in minimal medium, yet the active bacteriocin was not detectable [32]. Therefore we investigated if erinacine C could reduce the amount of mutacins produced by *S. mutans.* We first determined the sensitivity of the sensor strains *Lactococcus lactis* and *S. sanguinis* against the three mutacins of *S. mutans*. To this end we constructed gene deletion mutants for each of the three mutacins and tested how growth of the sensor strains *L. lactis* and *S. sanguinis* was influenced by these mutants. Additional file 5 shows that *L. lactis* was killed mainly by mutacin V and *S. sanguinis* mainly by mutacin IV, as expected. Deletion of SMU.423 had no influence on the zone of inhibition of both strains, confirming that mutacin VI is either not produced, or is not active against these two sensor strains.

**Fig. 3:**
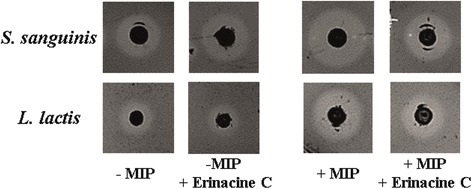
Erinacine C protects indicator strains *Lactococcus lactis* and *S. sanguinis* from killing by *S. mutans*. Overnight cultures of *S. mutans* UA159 in THBY (with and without MIP or erinacine C) were spotted on THBY plates and incubated for 4–6 h. Then, the plates were overlaid with exponential phase cultures of indicator strains grown in THBY and suspended in soft agar (0.7%). After 20 h of incubation (37 °C, 5% CO_2_) the area of zone of inhibition was measured. The image is a compilation of single technical replicate (cropped from the incubated agar plates) for the indicator strains and compound treatment after 20 h incubation

Finally we incubated the wild-type *S. mutans* UA159 with erinacine C. Figure 3 shows an example for the area of inhibition, and Table 2 shows the data from all experiments. The area of inhibition for *S. sanguinis* was reduced from 162 ± 24 mm^2^ to 106 ± 9 mm^2^ in the presence of erinacine C, representing 35% reduction. The effect on *L. lactis*, indicator for the highly expressed mutacin V, was even stronger (42% reduction of the area of inhibition). In the presence of MIP, the inhibition areas increased for both sensor strains as expected. Interestingly, addition of MIP increased the effect of erinacine C: If both erinacine C and MIP were applied, the area of inhibition was reduced by 47% for *S. sanguinis* and by 61% for *L. lactis*. The data show that erinacine C not only inhibited the transcription of mutacins, but the amount of the mature, active mutacin antibiotics in the cultivation medium was reduced in comparison to the untreated *S. mutans* UA159 culture. As a result, killing of the sensor strains was reduced. We hypothesize that erinacine C might similarly inhibit the synthesis of mutacins in dental plaque. *S. mutans* would therefore be less dangerous to competing bacteria and have a reduced fitness. The findings of our study support future studies aimed at evaluating the loss of virulence in a plaque/biofilm study.

**Table 2:** Reduced killing of mutacin IV and V indicator strains in the presence of erinacine C

	Treatment			
Indicator strain	control	+ Erinacine C	+ MIP	+ Erinacine C + MIP
*Streptococcus sanguinis*	162 ± 24	106 ± 9	239 ± 31	128 ± 12
*Lactococcus lactis*	121 ± 13	71 ± 8	168 ± 24	66 ± 6

Erinacine C was provided to *S. mutans* at 5 μg/ml final concentration, and MIP at 2.5 μg/ml final concentration. *S. sanguinis* is a specific indicator stain for mutacin IV, and *Lactococcus lactis* for mutacin V. See Methods for details on overlay assay. The area of the zone of inhibition was calculated and the data are mean and standard deviation of three biological replicates

## Discussion

Pharmaceutical industry has in the past routinely been using screens based on purified enzymes in high-throughput, triggered by comparative genomics which allowed identifying potential drug targets [40]. Although these screens can be automatized and used for testing huge synthetic libraries, the number of hits that were able to act on whole cells was disappointingly small (e.g. [41]). Using whole-cell screens has the advantage of finding only those molecules that are active against live cells. The disadvantage is that such screens are not easily automated and miniaturized, results depend on the physiological state of the cells, and most importantly, the mode of action of the compound and its molecular target remain unknown.

The seven compounds discovered here to inhibit mutacin transcription belong to completely different chemical classes of secondary metabolites (Table 1), thus they most probably reduce transcription of mutacins by different mechanisms. They also have entirely different modes of action: Noricumazol is a cation channel blocker [42], myxovirescin interferes with cell-wall synthesis [43], aurachin blocks the respiratory chain [44], and myxopyronin is an inhibitor of RNA polymerase [45]. The modes of action are unknown for chondrochloren and jerangolid, although the latter has been reported to change membrane permeability of the yeast *Hansenula anomala* [46]. The activity of the compounds on the mutacin reporter strains therefore most likely represents a secondary effect and the exact mechanism would have to be determined.

Erinacines are cyathane diterpenes produced by mycelial cultures of the edible and medicinal mushroom *Hericium erinaceus* and were originally reported to stimulate nerve growth factor biosynthesis [47]. The fruiting bodies as well as the mycelia of this species have been studied intensively in the past decades, and the most important findings were recently compiled in a review [48]. The compounds tested in the current study were derived from a project that recently resulted in the discovery of the novel corallocins from the related species, *Hericium coralloides* [49]. However, these neurotropic metabolites, as well as several other metabolites obtained from cultures and basidiomata of *Hericium* species, were found devoid of activities in the present study. Erinacine C is presently being prepared in large quantities by fermentation to further elucidate its biochemical mode of action and to further characterise its antiinfective and neurotropic activity.

In this study, erinacine C was demonstrated to suppress mutacin synthesis in *S. mutans* which defines a new biological activity for this molecule. No other study on the activity of erinacine C on bacteria was found, except one which reported a weak inhibition of *S. mutans* by extracts from *H. erinaceus* in a disc diffusion assay [50]. To show that reduction of mutacin biosynthesis by erinacine C reduces the fitness of *S. mutans* in vivo, it would be necessary to study the effect of this compound on the survival of *S. mutans* in multi-species oral biofilm models [51] or dental plaque communities.

Interestingly, erinacine C reduced the transcription of all three mutacins of *S. mutans*, and synthesis and maturation of at least two of them. Therefore the target of erinacine C must be upstream of the individual synthesis genes and common for all three mutacins. The mutacin synthesis in *S. mutans* is regulated by the two-component system ComDE and the quorum sensing signal MIP (mutacin inducing peptide) [32]. The signaling molecule MIP is secreted into the environment, and after reaching a certain concentration binds to the transmembrane histidine kinase ComD. This leads to the phosphorylation of its cognate cytoplasmic response regulator ComE. Transcription of all three mutacins is initiated by binding of phosphorylated ComE to specific promoter regions in the bacteriocin genes. Since transcription of all three mutacins was supressed by erinacine C, we hypothesize that this compound interacted with the ComDE signalling cascade. It would be interesting to investigate this hypothesis to see if erinacine C interferes with ComDE of *S. mutans*, and if it possibly interferes with two-component systems of other bacteria, which would make it an interesting anti-virulence molecule. Previously walkmycin, a complex secondary metabolite from *Streptomyces* sp., was shown to inhibit autophosphorylation of histidine kinases of *S. mutans* UA159 and thus reduce its virulence [52, 53].

## Conclusion

In this study, we demonstrated that a reporter strain for a gene that is important in terms of the ecology of the organism can be a useful screening tool. The observed reduction of mutacin synthesis might impair the survival of *S. mutans* in dental plaque and thus its virulence, but further studies are needed to show this. The strategy could be applied to other pathogens and other genes of interest.

## Additional files

Additional file 1: Primers used in this study and their use. (DOCX 16 kb)

Additional file 2: Growth of reporter strains *S. mutans* 150p, P1914p and 423p (with *gfp+*). *gfp +* tagged reporter strains for 150p (A), 1914p (B) and 423p (C) were cultivated in BM medium on a microtitre plate at 37 °C, 5% CO_2_ and monitored for growth (OD _620_) using a VICTOR plate reader from 0 to 8 h and 24 h. Data represent the mean and standard deviation of two biological replicates which were conducted with triplicate subsamples. (TIFF 893 kb)

Additional file 3: Growth of *S. mutans* UA159 Δ423. Knock-out strains for mutacin encoding genes were created by replacing genes with erythromycin B cassette and the growth (OD _600_) was monitored in THBY. Data show the mean and standard deviation of a biological replicate which were conducted with triplicate subsamples. (TIFF 848 kb)

Additional file 4: Concentration dependent inhibition of mutacin transcription by erinacine C. Reporter strains for bacteriocin gene expression 150p, 1914p and 423p were cultivated as in Fig. 1, and erinacine C was added at the indicated final concentrations. Growth and fluorescence are shown in % of the control (reporter strain induced by MIP). Data show mean and standard deviation of two biological replicates which were conducted with triplicate subsamples. (TIFF 1151 kb)

Additional file 5: Mutacin overlay assay to determine the specificity of the sensor strains. Overnight cultures of wild**-**type and knock-out strains for mutacins of *S. mutans* UA159 were spotted on THBY agar and allowed to incubate for 4–6 h. The exponential cultures of indicator strains *S. sanguinis* and *L. lactis* were overlaid on the plates using 0.7% agar. The area of zone of inhibition was measured after 20 h of incubation. (TIFF 1398 kb)

## Abbreviations

AHL: acylated homoserine lactone
AMA: Aspergillomarasmine A.
BM: Biofilm medium
DMSO: Dimethyl-sulfoxide
DNA: Ddesoxyribonucleic acid
GFP: Green fluorescent protein
HZI: Helmholtz-Center for Infection Research
KOM: Natural compound library from the research group “Microbial Communication” (KOM) at the Helmholtz-Center for Infection Research
LB: Luria Bertani
MIP: Mutacin inducing peptide
MWIS: Natural compound library from the department Microbial Drugs (MWIS), Helmholtz-Center for Infection Research, Germany
NDM-1: Metalloproteinase
OD: Optical density
PCR: Polymerase chain reaction
rpm: Rounds per minute
RT: Room temperature
SAAR: Natural compound library from the Helmholtz-Institute of Pharmaceutical Research, Saarbrücken, Germany
SOC: Medium used in the last step of transformation
THBY: Todd Hewitt broth supplemented with 0.5% (*w*/*v*) yeast extract
TSS: Buffer to make chemically competent cells
VICTOR: Multilabel fluorescent plate reader
