# Supplementary material for: Screening for inhibitors of mutacin synthesis in Streptococcus mutans using fluorescent reporter strains
Source: BMC Microbiol. 2018 Mar 27;18:24. doi: 10.1186/s12866-018-1170-3 (PMC5870221; doi:10.1186/s12866-018-1170-3)
Supplement: Supplementary file 1 — Primers used in this study and their use. (DOCX 16 kb) [file 12866_2018_1170_MOESM1_ESM.docx]

**Primers used in this study and their purpose of use.**

| **Sequence** | **Purpose** |
| --- | --- |
| TCGAATTCGCGGCCGCGCTAGCTTTGATAAGCATGCGAACTTAAAATACAAATATGG | Amplification of promoter of SMU.150 |
| AAAGTTCTTCTCCTTTTGTATCCATATGATAAACACCCCTTTTTCA | Amplification of promoter of SMU.150 |
| TCGAATTCGCGGCCGCGCTAGCTCTGTTAAACAGCCGGAAAAATGTTGA | Amplification of promoter of SMU.1914 |
| AAAGTTCTTCTCCTTTTGTATTCATATGATAAATACCCCTTCC | Amplification of promoter of SMU.1914 |
| TCGAATTCGCGGCCGCGCTAGCTATAAAATTCCCGATTTAACTTTTATC | Amplification of promoter of SMU.423 |
| AAAGTTCTTCTCCTTTTGTATTCATATGATAGATACCTCTTTTCC | Amplification of promoter of SMU.423 |
| TTCCCCGCCACTACTAAACA | Deletion of SMU.150 |
| GGCGCGCCAATGAAAGTGTTTGGCTGTCCA | Deletion of SMU.150 |
| GGCCGGCCAGGCACCCATATATTCTTGCTG | Deletion of SMU.150 |
| TTGAGACGTACCACCACTGC | Deletion of SMU.150 |
| TGAGCGAATGAAGTGAGCTTT | Deletion of SMU.423 |
| GGCGCGCCTCGTTAAATTGTTCAAATGCTTG | Deletion of SMU.423 |
| GGCCGGCCTAGGTCTGGAGCAGCTGTAGGT | Deletion of SMU.423 |
| CTGCGCTTTCAATACCGTCT | Deletion of SMU.423 |
| ACTGCCTGAGATGGAGTTGC | Deletion of SMU.1914 |
| GGCGCGCCTCGTTAAATTGTTCAAATGCTTG | Deletion of SMU.1914 |
| GGCCGGCCCGGAGGAGCTCTTAATTCCTG | Deletion of SMU.1914 |
| TCTTTTTGTTTTAGAAACTTCTGCT | Deletion of SMU.1914 |
