# Supplementary figures and images for: Screening for inhibitors of mutacin synthesis in Streptococcus mutans using fluorescent reporter strains
Source: BMC Microbiol. 2018 Mar 27;18:24. doi: 10.1186/s12866-018-1170-3 (PMC5870221; doi:10.1186/s12866-018-1170-3)

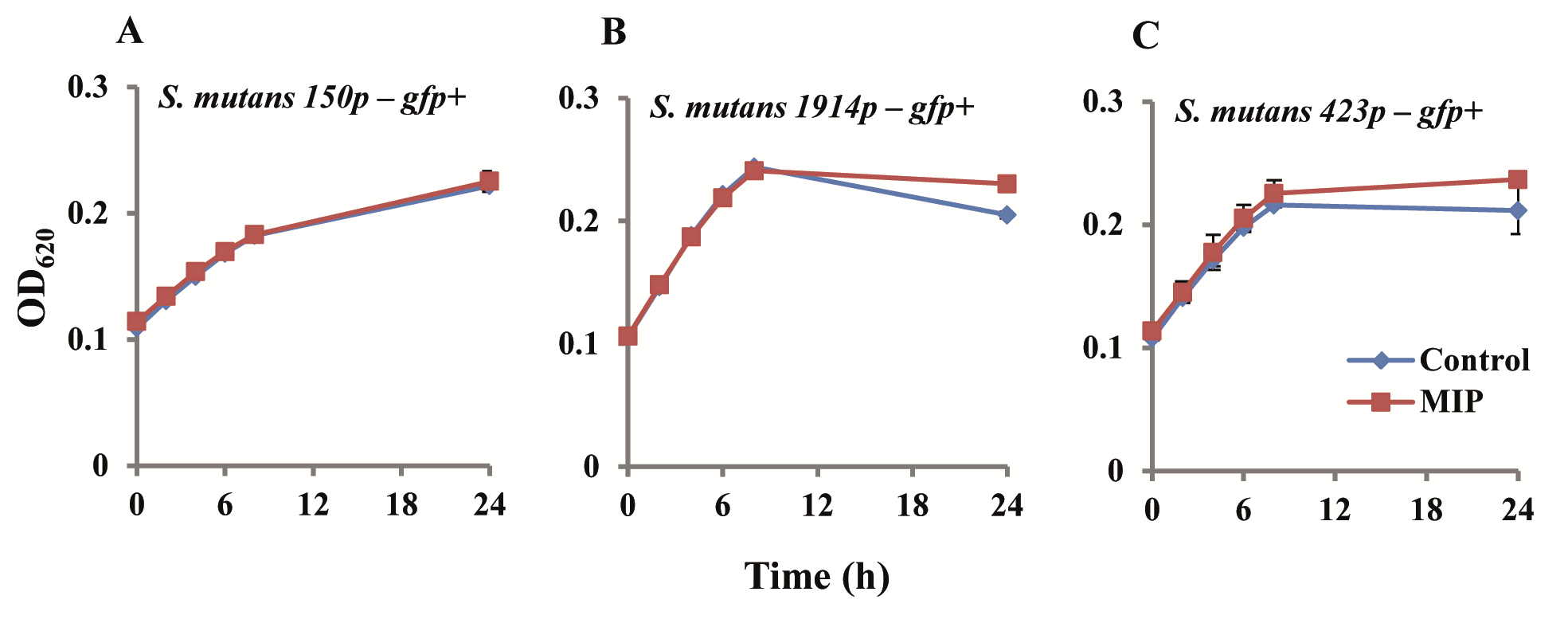

Supplement: Supplementary file 2 — Growth of reporter strains S. mutans 150p, P1914p and 423p (with gfp+). gfp + tagged reporter strains for 150p (A), 1914p (B) and 423p (C) were cultivated in BM medium on a microtitre plate at 37 °C, 5% CO2 and monitored for growth (OD 620) using a VICTOR plate reader from 0 to 8 h and 24 h. Data represent the mean and standard deviation of two biological replicates which were conducted with triplicate subsamples. (TIFF 893 kb) [file 12866_2018_1170_MOESM2_ESM.tif]

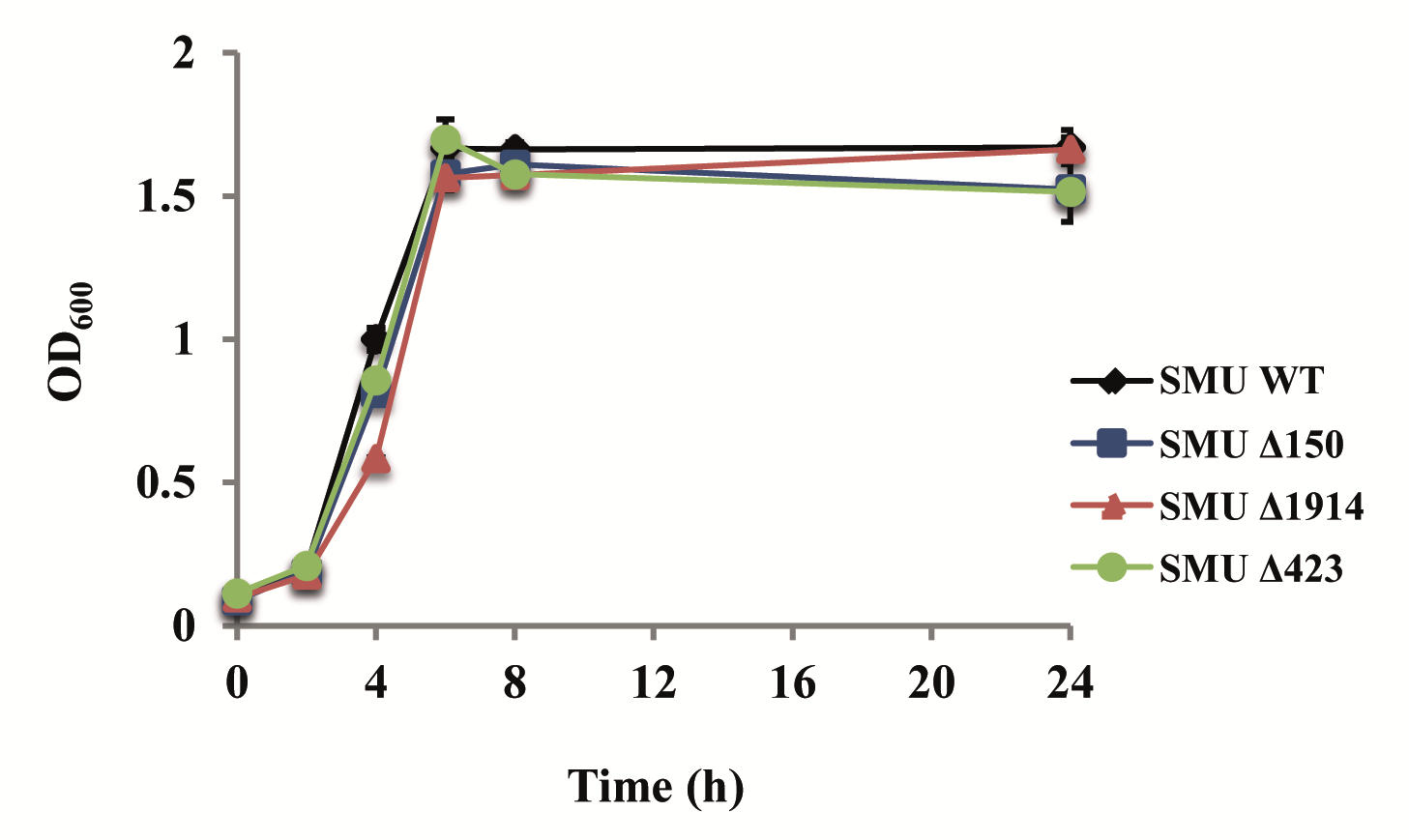

Supplement: Supplementary file 3 — Growth of S. mutans UA159 Δ423. Knock-out strains for mutacin encoding genes were created by replacing genes with erythromycin B cassette and the growth (OD 600) was monitored in THBY. Data show the mean and standard deviation of a biological replicate which were conducted with triplicate subsamples. (TIFF 848 kb) [file 12866_2018_1170_MOESM3_ESM.tif]

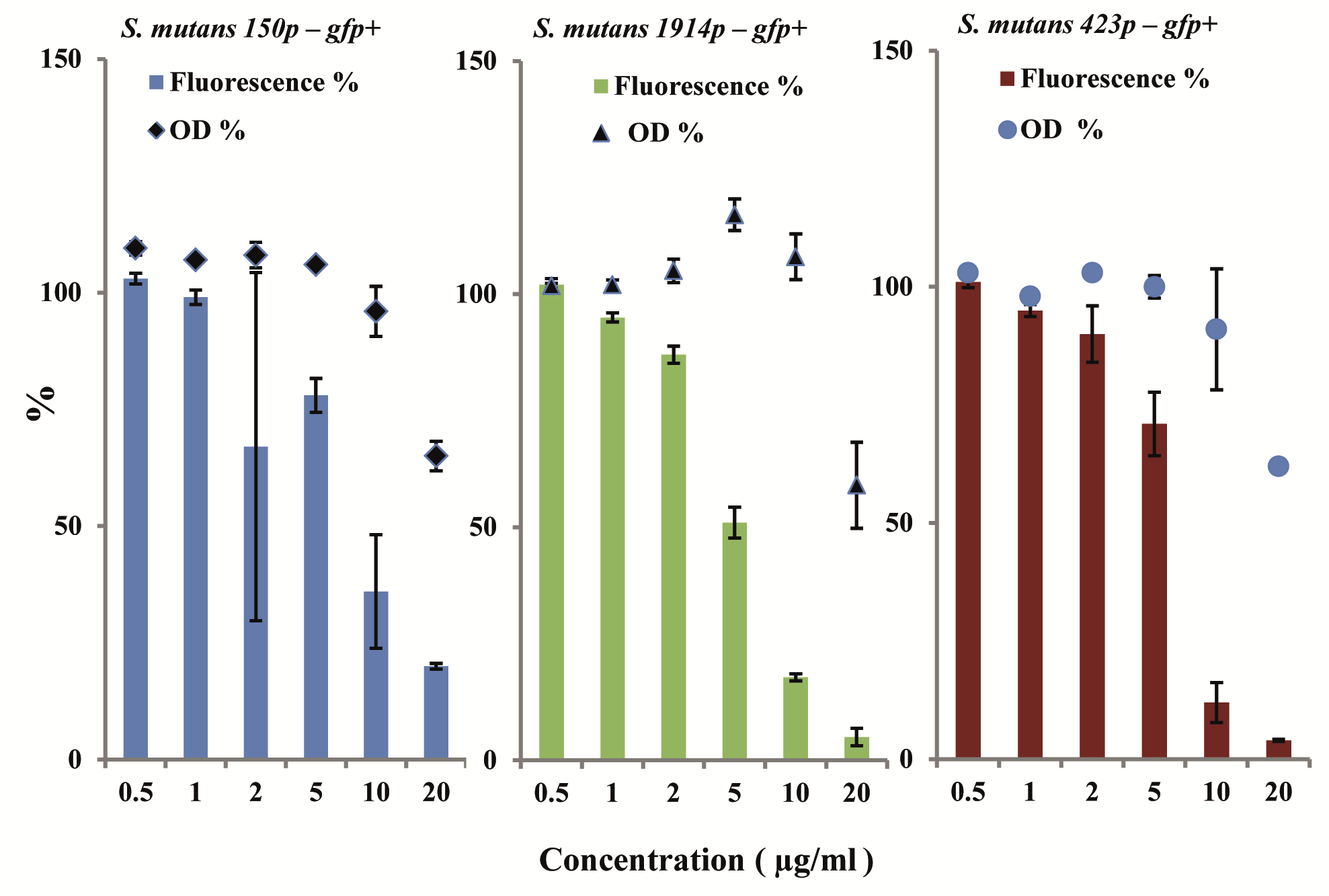

Supplement: Supplementary file 4 — Concentration dependent inhibition of mutacin transcription by erinacine C. Reporter strains for bacteriocin gene expression 150p, 1914p and 423p were cultivated as in Fig. 1, and erinacine C was added at the indicated final concentrations. Growth and fluorescence are shown in % of the control (reporter strain induced by MIP). Data show mean and standard deviation of two biological replicates which were conducted with triplicate subsamples. (TIFF 1151 kb) [file 12866_2018_1170_MOESM4_ESM.tif]

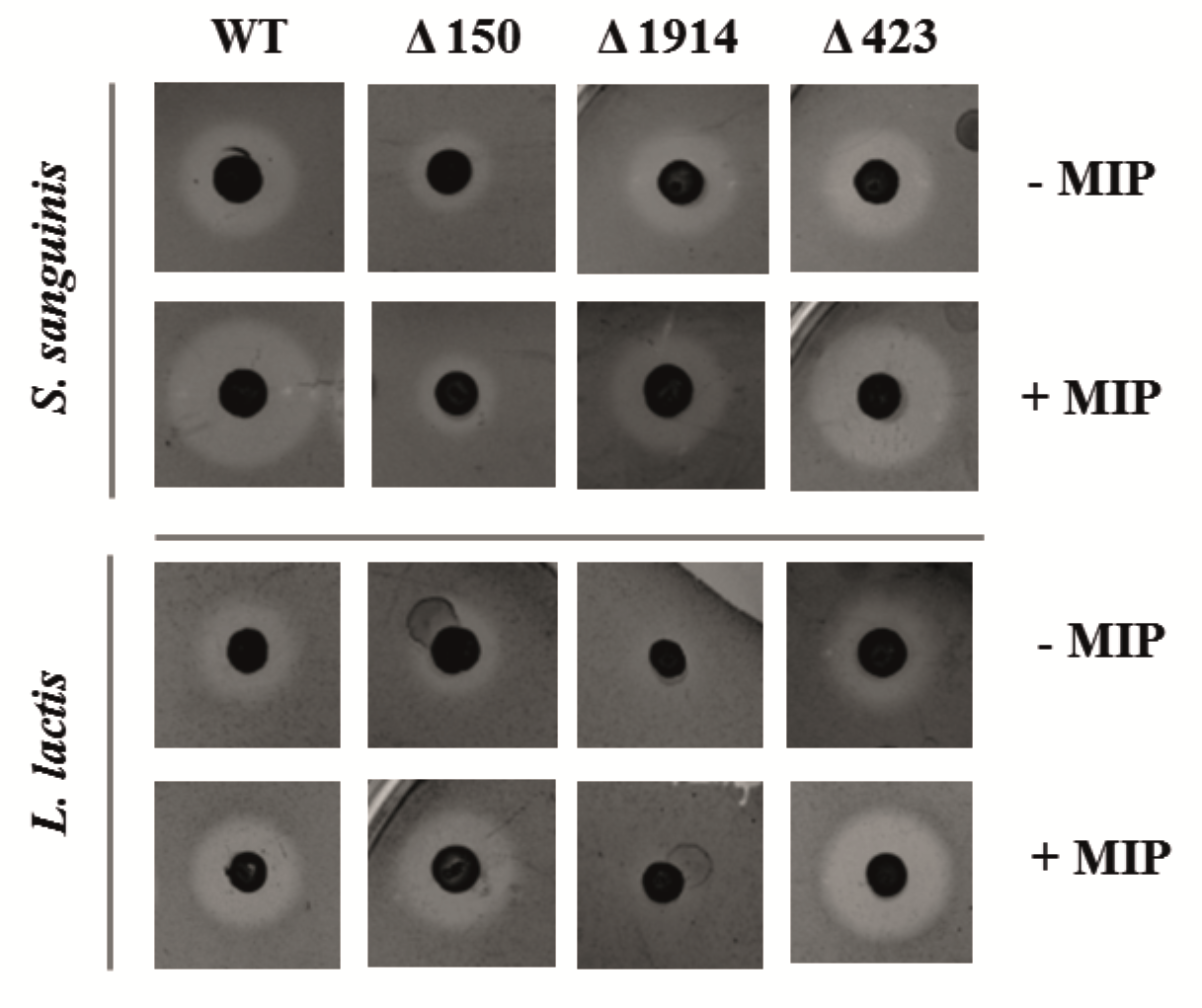

Supplement: Supplementary file 5 — Mutacin overlay assay to determine the specificity of the sensor strains. Overnight cultures of wild-type and knock-out strains for mutacins of S. mutans UA159 were spotted on THBY agar and allowed to incubate for 4–6 h. The exponential cultures of indicator strains S. sanguinis and L. lactis were overlaid on the plates using 0.7% agar. The area of zone of inhibition was measured after 20 h of incubation. (TIFF 1398 kb) [file 12866_2018_1170_MOESM5_ESM.tif]
